# A customisable pipeline for the semi-automated discovery of online activists and social campaigns on Twitter

**DOI:** 10.1007/s11280-021-00887-2

**Published:** 2021-06-11

**Authors:** Flavio Primo, Alexander Romanovsky, Rafael de Mello, Alessandro Garcia, Paolo Missier

**Affiliations:** 1 School of Computing, Newcastle University, Science Central, Newcastle upon Tyne, UK; 2 PUC-Rio/ CEFET-RJ, Rio de Janeiro, Brasil; 3 PUC-Rio, Rio de Janeiro, Brasil

**Keywords:** Twitter analytics, Online user discovery, Online activists, Online influencers, Influence theories

## Abstract

Substantial research is available on detecting *influencers* on social media platforms. In contrast, comparatively few studies exists on the role of *online activists*, defined informally as users who actively participate in socially-minded online campaigns. Automatically discovering activists who can potentially be approached by organisations that promote social campaigns is important, but not easy, as they are typically active only locally, and, unlike influencers, they are not central to large social media networks. We make the hypothesis that such interesting users can be found on Twitter within temporally and spatially localised *contexts*. We define these as small but topical fragments of the network, containing interactions about social events or campaigns with a significant online footprint. To explore this hypothesis, we have designed an iterative discovery pipeline consisting of two alternating phases of user discovery and context discovery. Multiple iterations of the pipeline result in a growing dataset of user profiles for activists, as well as growing set of online social contexts. This mode of exploration differs significantly from prior techniques that focus on influencers, and presents unique challenges because of the weak online signal available to detect activists. The paper describes the design and implementation of the pipeline as a customisable software framework, where user-defined operational definitions of online activism can be explored. We present an empirical evaluation on two extensive case studies, one concerning healthcare-related campaigns in the UK during 2018, the other related to online activism in Italy during the COVID-19 pandemic.

## Introduction

Online activists are individuals or organisations, that demonstrate an inclination to become engaged in social issues by participating in online social campaigns, often across a range of topics. These form an important class of online users, who may be particularly sensitive to requests for help on specific issues from, for instance, third sector organisations or government agencies. Reliably detecting activists is therefore an interesting goal. For example, in our prior work we described efforts to support health officers in tropical countries, specifically in Brazil, in their fight against virus epidemics like Dengue and Zika. Help from community activists is badly needed to supplement the scarce public resources deployed on the ground, and efforts have been recorded to document how technology can be brought to bear for this [3, 29]. Our own work in this setting has so far focused on identifying relevant content on Twitter that may point health authorities directly to mosquito breeding sites [31], as well as to users who have shown interest in those topics, i.e., by posting relevant content on Twitter [20]. In this work we generalise such efforts, with an aim to develop techniques for the semi-automatic detection of online activists on Twitter.

We start from the definition of *activist* according to the Cambridge Dictionary, namely “A person who believes strongly in political or social change and takes part in activities such as public protests to try to make this happen”. While activism is well-documented, e.g. in the social movement literature [7], and online activism is a well-known phenomenon [19], research has been limited to the study of its broad societal impact. In contrast, we are interested in the fine-grained discovery of activists at the level of the single individual. The presence of activists in social media is widely acknowledged, and it is also clear that social media facilitates activists communication and organisation [24, 33]. Specific traits that characterise activists include awareness of causes and social topic and the organisation of social gatherings and activities, including in emergency situations, by helping organise support efforts and diffusion of useful information.

The two case studies used for validation in this work also serve as motivating examples. The first concerns UK online Health campaigns, where our goal is to identify Twitter users who are not necessarily known influencers, that is, they are not known for propagating information about the campaign, but instead are actively engaged with online conversations about the issues. Amongst the top-10 ranked users accounts discovered using our pipeline (see Table 4), 50% to 90% (depending on the ranking metric chosen) are for individuals who are not institutionally associated with the campaigns. In contrast, influencers in this example would be organisations such as the UK’s National Health Service or the Ministry of Health. The second case study is also in the Health domain and includes grassroots as well as institutional initiatives related to COVID-19 in early 2020. Again, the top-10 users (Table 9 are individuals who stand out because of their engagement with the campaigns, rather than by virtue of spreading information about them.

While in these examples activists are fairly well-defined, in general the notion is not as well formalised in the literature as that of, for example, *influencers*. Thus, our approach is to develop a *configurable* content processing pipeline which can be customised to identify a variety of classes of users. The pipeline repeatedly searches for and ranks Twitter user profiles by collecting quantitative network- and content-based user metrics. Once targeted to a specific topic, it provides a tool for exploring operational definitions of user roles, including online activism, i.e., by combining the metrics into higher level, *engineered* user features to be used for ranking. Furthermore, top-ranking users are automatically followed in the hope that they keep showing engagement with other socially relevant online topics. These are then analysed semi-automatically to discover new social contexts, where new users can in turn be found.

To be clear, this work is not about providing a robust definition of online activism, or to demonstrate that online activism translates into actual engagement in the “real world”. Instead, it allows researchers, including social scientists, to experiment with multiple specific definitions of activism through the automation of most of the data harvesting and user discovery process.

### Challenges

The potentially more subdue nature of activists, relative to that of influencers, makes it difficult to distinguish the online footprint of activists from the background noise resulting from generic conversations. Also, we observe that activists are by their nature associated with specific topics and, manifesting it through their engagement in *local* contexts, as opposed to influencers who are naturally interested in spreading information at a global level. Finally, identifying activists requires temporal continuity of demonstrated engagement. The combination of these elements translate into a number of technical challenges as models and algorithms developed for influencers [16, 17], such as those surveyed in Section 2 are not immediate applicable, because those tend to operate on global networks.

Specifically, a number of topic-sensitive metrics and models have been proposed to measure social influence, for example, *alpha centrality* [8, 22] and the *Information Diffusion* model [23]. Algorithms based on topic models have also been proposed to account for topic specificity [35]. However, these approaches are still aimed at measuring influence, not activism. They also assume a one-shot discovery process, as opposed to the continuous, incremental approach proposed in this work.

In contrast, in our approach we start from the assumption that *social contexts can be represented as collection of hashtags*. Thus, discovering new contexts entails finding sets of hashtags that are used consistently together by a sufficient number of users. This is difficult because of widespread noise and inconsistencies in hashtag usage, as well as other known problems such as synonym hashtags. Furthermore, contexts are by their nature temporally localised, however establishing proper temporal boundaries is difficult when events have “lead up” and “climb down” phases. Finally, we have chosen not to rely on external semantic knowledge around hashtags, and instead apply clustering algorithms to discover similarities amongst groups of hashtags.

### Approach and contributions

Our main contribution is the *design, implementation, and empirical evaluation of an iterative user and context discovery pipeline*. When executed over time and across multiple iterations, the pipeline produces an ever-growing database of user profile features, which can then be used for mining purposes.

The approach consists of two phases, as follows. Let us assume that an initial set of contexts is given. These are topic-specific and limited both in time and, optionally, also in space, i.e., regional initiatives, events, or campaigns. In the *first phase*, we search for users only within these contexts, following the intuition that low-key users who produce weak online signal have a better chance to be discovered when the search is localised and then repeated across multiple such contexts. We then collect a number of network-based and content-based user profile features, mostly known from the literature, and make them available to user-defined user ranking functions.

In the *second phase*, we follow the (public) online history of top-ranked users and conduct a hashtag analysis aimed at discovering new contexts. We automatically generate user communities from user interactions as well as from hashtag co-occurrence in posts, and select hashtags that are relevant within those communities, whenever possible. We also perform peak analysis on hashtag usage to identify temporal boundaries. The resulting candidate contexts are then manually inspected and selected for semantic relevance.

We provide an empirical evaluation consisting of two case studies to evaluate the feasibility of our approach. In the first case study, we collected about 3 500 users across 25 contexts in the domain of healthcare awareness campaigns in the UK during 2018, and demonstrated the application of three choices of ranking functions, showing that it is possible to identify individuals as opposed to well-known organisations, and to discover new follow-up contexts, which in this case are relevant previously unknown social events with a definite Twitter footprint.

In the second case study, we explored contexts around the 2020 COVID crisis with specific focus on the Italian Twitter population. We found that, given 24 seed contexts, we successfully discovered over 3 000 users who qualify as activists according to our definition. The top-100 ranked users consist almost entirely (96/100) of individuals, as opposed to well-known organisations, confirming that our strategy is useful to discover new an unexpected knowledge in the network. Of these, 45 are *on topic*, i.e., they are effectively focused on the social campaigns we used as initial contexts. Furthermore, we also identified 192 new contexts, of which 24 are relevant and previously unknown social campaigns.

This paper substantially extends [25], specifically adding: (i) semi-automated context discovery, and (ii) its empirical evaluation through a new case study on activism as it relates to the recent (as of 2020) COVID-19 crisis.

## Related work

The closest body of research to this work is concerned with techniques for the discovery of online *influencers*. According to [15], influencers are *prominent individuals with special characteristics that enable them to affect a disproportionately large number of their peers with their actions*. A large number of metrics and techniques have been proposed to operationalize this generic definition [27]. These metrics and techniques tend to favour high visibility users across global networks, regardless of their actual impact [11]. In contrast, activists are typically low-key, less prominent users who only emerge from the crowd by signaling high levels of engagement with one or more specific topics, as opposed to being thought-leaders.

### Identifying influencers and prominent users

Despite this conceptual difference, research efforts addressing online influencers deserves special attention. Although we may describe the behaviour of online influencers by using well-tested metrics [27], different approaches and techniques have been proposed for properly identifying and ranking online influencers in different contexts. A method for creating Twitter users’ ontologies based on the content type of their tweets is proposed in [26]. This approach could be used to gain insights over a user, but due in part to Twitter API limitations, it is limited to recent posts. Consequently, it fails to provide a comprehensive description of a user’s activity.

The algorithm proposed in [9] aims at identifying influencers based on relevant social media conversations from a single topic context. The authors use a set of metrics, including the number of “likes”, the number of viewers per month, frequency of posts, the number of comments per post, and the ratio between positive and negative posts. This approach is not easy to automate, as some of these metrics are qualitatively gathered and difficult to acquire. Another approach to ranking topic-specific influencers in the context of specific events appears in [15]. The authors propose accounting network dynamics in real-time. However, the effect of these works was to discover users who receive much attention, which does not necessarily result in an effective impact on users from a particular topic. Unlike the majority of the influencer ranking algorithms, Schenk et al. [30] propose a topic-specific influencer ranking. First, it harvests sequentially timed snapshots of the network of users addressing the topic. Then, it ranks the users based on the number of followers gained and lost in the considered snapshots.

Machine learning techniques have been also used for identifying and ranking influencers. In [4], machine learning is used to analyse posted content and recognise when users can influence others during a conversation. However, this approach requires composing a substantial ground truth *a priori*, making it unfeasible for our purposes. Besides, the need to create a classifier for each topic limits the scalability of the system. Similarly, a supervised regression approach is used in [21] to rank the influence of Twitter users. This approach uses features not based on content, but the authors concluded that the method performs poorly as it requires a huge training set to work effectively.

Alternatively to the concept of influencers, [6] presents a model for identifying “prominent users” regarding a specific topic event on Twitter. For the authors, prominent users are those who focus their attention and communication on the aforementioned topic event. This model describes users through a feature vector computed in real-time, which allows a separation between on-topic and off-topic users’ activity over Twitter. However, similar to [4], problems of scalability and adaptability arise once two supervised learning methods are used: one to distinguishing prominent users from the rest and the other to rank them.

### Identifying groups of users

In some cases, such as for supporting marketing campaigns, optimising the influence of the campaign over particular geographical regions is a need. In this way, Li et al. [17] formally define the problem of maximum geographic spanning regions (MGSR) over location-aware social networks, proposing a greedy algorithm to solve the problem. By this approach, users may identify the best top-*k* sets of seeds, which maximally influences the users’ preferred regions. However, Cai et al. [10] argue that the influence maximisation should also take into account the opportunity of users influencing each other through physical interactions. For this purpose, the authors formulate the HIM (holistic influence maximisation) query problem. The HIM problem is based on a holistic influence spread model combining social connection, spatial connection, and preference-based similarity connection. Another great concern of certain campaigns would be influencing as many communities as possible from the same seeds. For such cases, Li et al. [16] propose a metric to measure the community-diversified influence, aiming to reach its maximisation. In this way, the authors propose heuristics for selecting local seeds and for performing iterative local searches of seed nodes.

There are also cases in which geo-social groups should be identified in a social network for performing impromptu activities. In such cases, it is common to expect that these groups should attend to multiple constraints, including users‘ skills and minimum group size. For this purpose, Chen et al. [12] propose a two-stage search framework for supporting the optimized discovery of geo-social groups satisfying multiple constraits.

With regards to geo-localisation, the approach taken in our work is different, because of the different characterisation of activists vs influencers. Namely, we work on the assumption that the identification of activists within specific contexts also works as a natural depictor of geospatial settings, as defined by the scope of the campaign. The contexts found in the empirical evaluations reported in this paper, for example, present clear examples of this association between activism and geolocation.

### Identifying contexts

Regarding the search for new contexts, the closest body of research to this work is concerned with techniques for retrospective event detection (*RED*) on Twitter [32], which focus on the discovery of previously unrecognised events from historical data instead of discovering new events in real-time. Several RED event detection algorithms for Twitter have been proposed [2]. An interesting approach consists in “feature-pivot” techniques which model an event over tweet streams as a bursty activity. Some features are characterised by an heightened frequency in correspondence of an event. The hypothesis made is that related words usage would increase as an event unfolds, this makes use feature distribution analysis and the grouping of features with similar frequency trends.

The algorithm proposed in [18] tackles effectively the problem of learning embedding of hashtags and tweets. It semantically cluster hashtags and tweets by exploiting a hierarchical embedding framework. It considers as features the co-occurrences of hashtags and words included in the tweets, but it does not recognise the role of the author of the tweets.

## Definitions

In this section, we provide a formal grounding for our work. It consists of a definition of *contexts* within which activists are to be found, and of a collection of Twitter-specific metrics to establish user relevance within a context. Clear examples to demonstrate the usefulness of these metrics for our purposes are given in Section 6, where they are applied to two separate case studies.

### Contexts and context networks

The central notion of a *context* is grounded in the familiar notion of a simple Twitter query, consisting of a set of search terms, extended to include spatio-temporal boundaries. Formally, a *context* *C* consists of a set *K* of hashtags and/or keyword terms, a time interval [*t*_1_,*t*_2_], and a geographical constraint *s*, such as a bounding box:

1 $$ C = (K, [t_{1}, t_{2}], s)  $$

Let *P*(*C*) denote the query result, i.e., a set of tweets made by users. We only consider two Twitter user activities: an *original tweet*, or a *retweet*, together with the contained user *mentions* from both. Let *u*(*p*) be the user who originated a tweet *p* ∈ *P*(*C*). We say that both *p* and *u*(*p*) are *within context* *C*. We also define the complement *P*~(*C*) of *P*(*C*) as the set of posts found using the same spatio-temporal constraints, but which do not contain any of the terms in *K*. More precisely, given a context $C^{\prime }= (s, [t_{1}, t_{2}], \emptyset )$ with no terms constraints, we define $\Tilde {P}(C) = P(C^{\prime }) \setminus P(C)$. We refer to these posts, and their respective users, as “out of context *C*”.

*P*(*C*) induces a user-user social network graph *G*_*C*_ = (*V*,*E*) where *V* is the set of all users who have authored any *p* ∈ *P*(*C*): *V* = {*u*(*p*)|*p* ∈ *P*(*C*)}, and a weighted directed edge *e* = 〈*u*_1_,*u*_2_,*w*〉 is added to *E* for each pair of posts *p*_1_,*p*_2_ such that *u*(*p*_1_) = *u*_1_,*u*(*p*_2_) = *u*_2_ and either (i) *p*_2_ is a retweet of *p*_1_, or (ii) *p*_1_ contains a mention of *u*_2_. For any such edge, *w* is a count of such pairs of posts occurring in *P*(*C*) for the same pair of users.

### User relevance metrics

As we seek to characterise activism as a specific user role within contexts, we borrow some of the well-established metrics that are available from recent research on social user roles in Twitter [27], for quantifying the relevance of a user within that context. These include topical focus, topical strength, topical attachment, follower rank and in-degree centrality.

These metrics are underpinned by a common set of basic features, which can be directly extracted from Twitter posts. Given a context *C* containing user *u*, these are defined as follows.

$$ \begin{array}{@{}rcl@{}} {R1}(u)& &\text{: Number of retweets by}\ u, \text{of tweets from other users in C;}\\ {R2}(u)&&\text{: Number of unique users in}\ C, \ \text{who have been retweeted by}\ u;\\ {R3}(u)&&\text{: Number of retweets of}\ u's \ \text{tweets;}\\ {R4}(u)&&\text{: Number of unique users in} \ C\ \text{who retweeted}\ u'\text{s\ tweets;}\\ {P1}(u)&&\text{: Number of original posts by}\ u\ \text{within}\ C;\\ {P2}(u)&&\text{: Number of web links found in original posts by}\ u\ \text{within}\ C; \\ {F1}(u)&& \text{: Number of followers of}\ u;\\ {F2}(u)&& \text{: Number of followees of}\ u \end{array} $$

Note that, given *C*, we can evaluate some of the features above with respect to either *P*(*C*) or *P*~(*C*) independently from each other, that is, we can consider an “on-context” and an “off-context” version of each feature, with the exception of *F*1 and *F*2 which are context-independent. For example, we are going to write *R*1_*on*_(*u*) to denote the number of context retweets and *R*1_*off*_(*u*) the number of out-of-context retweets by *u*, i.e., these are retweets that occur within *C*’s spatio-temporal boundaries, but do not contain any of the hashtags or keywords that define *C*. We similarly qualify all other features.

Using these core features, we can derive all five metrics mentioned above. Specifically, Topical Focus, Topical Strength, and Topical Attachment are *content-based* metrics that rely solely on content and require no knowledge of the user-user network. When considered relative to a topic of interest, i.e., a context, they are defined as follows (citations refer to literature where these metrics are defined).

2 $$ \begin{array}{@{}rcl@{}} {T{}opical F{}ocus~[21]:} ~{TF}(u) & = & \frac{{P1}_{{on}}(u)}{{P1}_{{off}}(u) +1} \end{array} $$

3 $$ \begin{array}{@{}rcl@{}} {T{}opical  Strength~[5]:} ~{TS}(u) & = & \frac{{P2}_{{on}}(u) \cdot \log({P2}_{{on}}(u) + R3_{{on}} + 1 )}{{P2}_{{off}}(u) \cdot \log({P2}_{{off}}(u) + R3_{{off}} + 1 ) + 1}  \end{array} $$

4 $$ \begin{array}{@{}rcl@{}} {T{}opical  Attachment~[7,25]:} ~{TA}(u) & = &\frac{{P1}_{{on}}(u) + {P2}_{{on}}(u)}{{P1}_{{off}}(u) + {P2}_{{off}}(u) +1} \end{array} $$

Each of these functions express the interest of a user towards a context by exploiting its tweets content. Given a user *u* who published tweets within the context’s spatio-temporal boundaries, these functions count the number of on-topic social interactions over the off-topic ones. In particular, (2) only considers the original posts published as a context’s interest measures, while (3) also takes into account the number of retweets and, together with (3), the published external links.

In contrast, Follower Rank and In-degree Centrality are *topological metrics* that encode context-independent long-lived relationships amongst users, i.e., follower/followee, and user relationships that occur specifically within a context, respectively:

5 $$ \begin{array}{@{}rcl@{}} {F{}ollower Rank:} \quad {FR}(u) = \frac{{F1}(u)}{{F1}(u)+{F2}(u)}  \end{array} $$

6 $$ \begin{array}{@{}rcl@{}} {I{}n{}-{}d{}e{}g{}r{}ee C{}e{}nt{}r{}al{}i{}t{}y:} \quad {IC}(u) = \frac{{indegree}(u)}{N-1} \end{array} $$

where *N* is the number of nodes in the network induced by *C*. Note that the metrics we have selected are a superset of those indicated in recent studies on online activism, namely [19] and [24], and thus support our empirical evaluation, described in Section 6.

## Activists discovery strategy

**Fig. 1:**
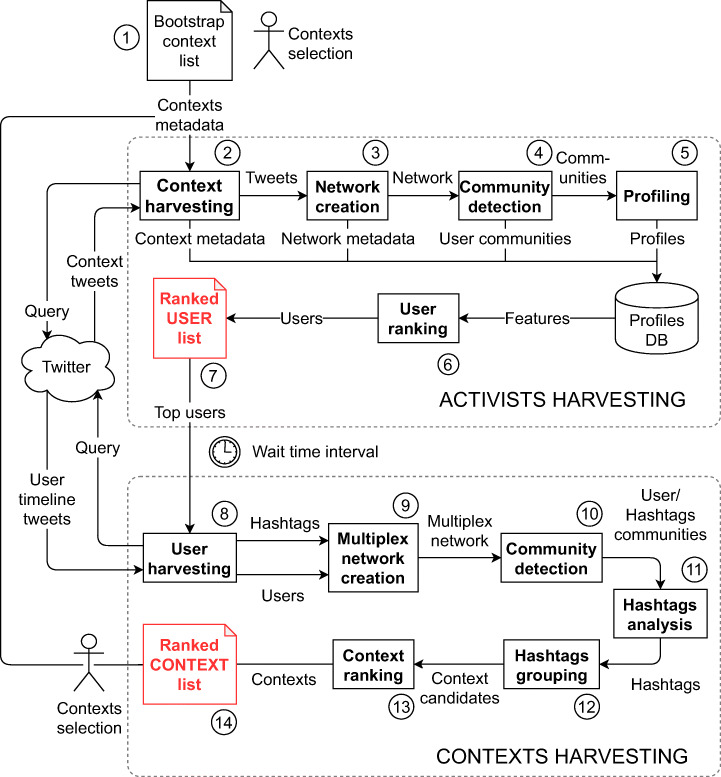
Schematic diagram of the user and context discovery phases. Note that an initial list *C* of contexts (events) is provided to initialise the whole pipeline (1). The profiles DB stores the ever growing list of users harvested at each iteration of the whole pipeline (with the exception of the very first run which starts with a bootstrap context list)

Our main contribution is the design and implementation of a configurable Twitter feed processing pipeline, shown in Figure 1, aimed at semi-automatically discovering online activists as defined above. We first describe the pipeline at a high level, and in the rest of the section we provide a technical account of its components.

The pipeline takes an initial set of contexts, defined as in Section 3.1 as spatio-temporal Twitter search queries and shown at the top of Figure 1, and through a series of iterations it incrementally produces a growing users profiles database of relevant online activists (*profile DB* in the figure).

**Fig. 2:**
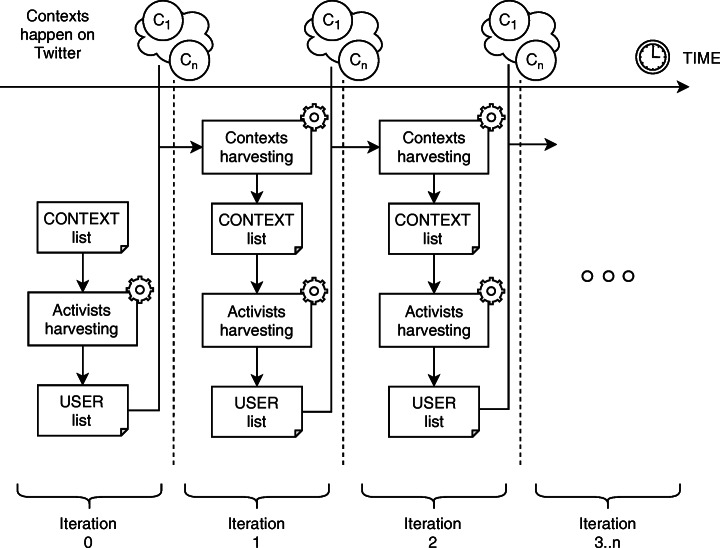
Each iteration of the pipeline is marked by a time interval [*t*_1_,*t*_2_]. In each interval, contexts occur in Twitter and are ready to be harvested on the successive pipeline iteration. The only exception is iteration 0, started from a bootstrap context list

Specifically, the pipeline implements an iterative strategy consisting of two interleaved phases: (1) discovery of new *activists* and (2) discovery of new *contexts*. The intuition behind this strategy is that some of the activist users who will have been involved in relevant contexts for a certain time duration, may continue to engage with similar issues. Thus, in this interleaving, illustrated in Figure 2, we analyse the content produced by the top activists *after* the events associated with the initial contexts, and use them to discover new contexts (2) that those same users may have been involved in.

In turn, new activists can then be found using the new contexts. These will typically be within the same topic area, providing logical continuity to the discovery process, however the search may also “drift” to new areas of interest if enough of the users from the previous iteration have interests that “cross over” to other domains.

## Technical approach

We now provide technical insight into each phase of the pipeline.

### Harvesting new activists

Initially, a *seed* context is hand-generated (step 1 in Figure 1) such as the *2018 UK health campaigns* used as part of our evaluation. The contexts produced at the end of one iteration of phase 2 are used to feed the next phase 1, as shown in Figure 2.

During the Context Harvest (step 2), all Twitter posts *P*(*C*) that satisfy *C* are retrieved, using the Twitter Search APIs. Note that this step hits the API service limitations imposed by Twitter. For this reason, in our evaluation we have limited our retrieval to 200 tweets/context. This is sufficient, considering that repeated users appear consistently in our evaluation (Section 6). Twitter API limitations can be overcome by either extending the harvesting time, or by choosing more recent contexts, as the Twitter API is more tolerant with recent tweets.

In step 3, a context network *G*_*C*_ is then generated, as specified in Section 3.1. The size of each network is largely determined by the nature of the context, and ranges between 140 and 400 users (avg 254, see Table 1).

**Table 1:** List of contexts used in the experiments along with network metrics

Context name	Period (2018)	Nodes	Edges	Density	Avg degree	Assortativity
16 days of action	11-25 / 12-10	396	349	0.002	1.8	− 0.1
Elf day	12-03 / 12-12	365	436	0.003	2.4	− 0.2
Dry january	01-01 / 01-31	235	234	0.004	2.0	− 0.3
Cervical cancer prevention week	01-21 / 01-27	209	192	0.004	1.8	− 0.1
Time to talk day	02-06 / 02-07	268	231	0.003	1.7	− 0.2
Eating disorder awareness week	02-25 / 03-03	256	241	0.004	1.9	− 0.2
Rare disease day	02-28 / 03-01	294	206	0.002	1.4	− 0.2
Ovarian cancer awareness month	03-01 / 03-31	215	202	0.004	1.9	− 0.4
Nutrition and hydration week	03-11 / 03-17	273	326	0.004	2.4	− 0.3
Brain awareness week	03-11 / 03-17	307	281	0.003	1.8	− 0.1
No smoking day	03-13 / 03-14	254	219	0.003	1.7	− 0.3
Epilepsy awareness purple day	03-26 / 03-27	306	252	0.003	1.6	− 0.2
Experience of care week	04-23 / 04-27	176	196	0.006	2.2	− 0.1
Brain injury week	05-01 / 05-31	238	306	0.005	2.6	− 0.1
Mental health awareness week	05-14 / 05-20	268	245	0.003	1.8	− 0.5
Dementia action week	05-21 / 05-31	300	300	0.003	2.0	− 0.0
Mnd awareness month	06-01 / 06-30	141	234	0.012	3.3	− 0.3
Wear purple for jia	06-01 / 06-30	165	245	0.009	3.0	− 0.5
Carers week	06-11 / 06-17	270	277	0.004	2.1	0.0
National dementia carers	09-09 / 09-10	184	177	0.005	1.9	− 0.2
Mens health week	06-11 / 06-17	264	214	0.003	1.6	− 0.2
Stress awareness day	11-07 / 11-08	293	209	0.002	1.4	− 0.2
National dyslexia week	10-01 / 10-07	229	235	0.004	2.1	− 0.2
Ocd awareness week	10-07 / 10-13	202	193	0.005	1.9	− 0.6
Jeans for genes day	09-21 / 09-22	246	325	0.005	2.6	− 0.2

The size of each network is largely determined by the nature of the context. In the contexts used in our case studies, we measured a number of users ranging between 140 and 400 (avg 254, see Table 1). This is entirely determined by the Twitter conversation volume around that context in terms of number of published tweets and retweets.

Next (step 4), *G*_*C*_ is partitioned into communities of users. The goal of this partitioning is to further narrow the scope when computing the network’s in-degree centrality (6), to enable weak-signal users to emerge relative to other more globally dominant users. We have experimented with two of the many algorithms for discovering virtual communities in social networks, namely DEMON [13] and Infomap [28]. Both are available in our implementation, but based on our experimental comparison (Section 6) we recommend the latter.

Comparing briefly the two approaches, DEMON is based on *ego networks* [1], and uses a label propagation algorithm to assign nodes to communities. Users may be assigned to multiple communities, an attractive feature when users are active in more than one community within the same context, i.e., a social event or a campaign. Label propagation is also a local method, translating into an efficient algorithm. In practice, however, in our experiments we found that for almost half of our context networks, DEMON actually fails to discover any communities. In contrast, Infomap forces each user into at most one community, but it generates non-empty communities in all cases. As some of those are very small, our implementation discards communities with fewer than 4 users (see Section 6). Once communities are identified, we calculate the in-degree centrality (6) for each node. If the node belongs to one of the communities, their centrality is calculated *relative to the community*, or relative to the entire network, otherwise.

### Computing user features and ranking

Next, the user metrics are computed from the network and the user features, as defined in Section 3.2 along with the *Follower Rank* (step 6). This is achieved through bulk retrieval of user profile information (step 5), namely the number of tweets, retweets, number of followers *F*1(*u*) and followees, *F*2(*u*), along with user name, web link, and bio. Computing the other metrics: *Topical Focus* (2), *Topical Strength* (3), *Topical Attachment* (4) also requires the entire user post history to be retrieved for the entire time interval defined by the context. These posts are then separated into *on-context* and *off-context*, denoted *P*(*C*) and *P*~(*C*) respectively, depending on whether they contain at least a hashtag related to the context or not. Similarly, a post that contains a link is a *link on-topic* if it contains both a link and a hashtag related to the context, and a *link off-topic* otherwise. We also calculate the number of retweets for every post, i.e., *R*1(*u*) and *R*3(*u*), which are required to compute *Topical Strength*.

All of these features are persisted to the users profiles database, which is made available for ranking purposes. The database enables user-defined ranking functions, which result in user ranking lists (step 6). Examples of these are given later in Section 6. This framework approach is consistent with the experimental nature of our search for *activists*, which requires exploring a variety of ranking functions.

### New contexts discovery

As mentioned at the start of the section, Phase 2 aims to discover new contexts, so that a new iteration can start again i.e., from step 2. The alternating phases are illustrated in Figure 2. We ensure that iterations do no overlap in time, as otherwise we risk re-discovering the same users and artificially ranking them higher than others (because ranking rewards continuity of engagement). The time windows used to define contexts may vary with the duration of the events, and in our evaluation we experiment with empirical settings.

**Fig. 3:**
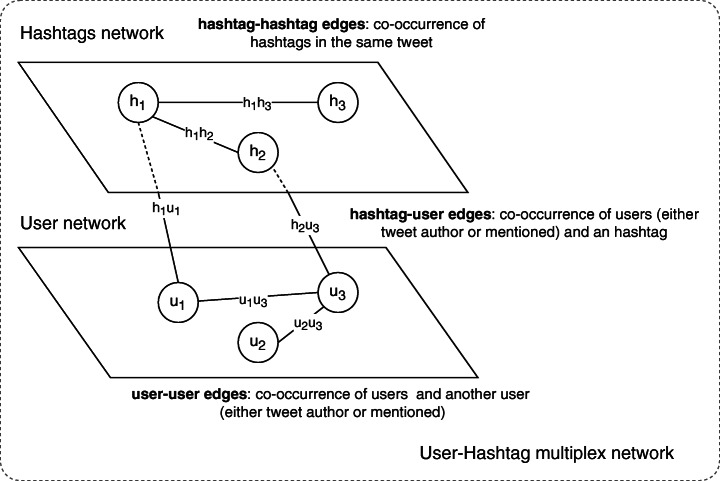
Schematic diagram of the hashtag-user multiplex weighted network. Note how the two different networks, composed by hashtag and user nodes, are connected with edges

Given a set *C*_*i*_ of bootstrap contexts (step 1) with time intervals [*t*_*im*_,*t*_*iM*_], the initial window is defined implicitly as $W = [t_{m}, t_{M}] = [\min \limits _{i} t_{im}, \max \limits _{i} t_{iM}]$. At the beginning of phase 2, the Twitter timelines for the top *k* users obtained at step (7) in the previous iteration are harvested for tweets published during *W*, resulting in a set of tweets *UT*_*W*_ (step 8). In step 9, these tweets are used to generate a weighted, non-directed, *multiplex network* graph. This is a two-layer network where the layers are interconnected as shown in Figure 3:

User-User network: a weighted, non-directed network *G*_*u*_ = (*V*_*u*_,*E*_*uu*_) where *V*_*u*_ is the set of all users appearing as either authors or mentioned in tweets from *UT*_*W*_. Edges *E*_*uu*_ connect users that co-occur in tweets as either author-mentioned or mentioned-mentioned relations, where the weights are the number of such relations for every node pair;
Hashtag-Hashtag network: a weighted, non-directed network *G*_*h*_ = (*V*_*h*_,*E*_*hh*_) where *V*_*h*_ is the set of all hashtags appearing in tweets from *UT*_*W*_. *E*_*hh*_ edges connect hashtags which co-occur in tweets, for which the weights are the number of such relations for every node pair.

The two layers are connected by an additional set of weighted edges *E*_*hu*_ which connect co-occurring users (either author or mentioned) and hashtags from *UT*_*W*_, where the weights are the number of such relations for every node pair. This construction results in a multiplex network *G*_*m*_ = (*V*_*m*_,*E*_*m*_) where *V*_*m*_ = *V*_*u*_ ∪ *V*_*h*_ and *E*_*m*_ = *E*_*hh*_ ∪ *E*_*uu*_ ∪ *E*_*hu*_.

In step 10, *G*_*m*_ is then partitioned into mixed communities of hashtags and users. The goal of this partitioning is to group related hashtags into topics, exploiting the relations between pairs of hashtags, and the network of users who publish them. The rationale behind this modelisation is the following: hashtags that co-occur the most must be somehow related to similar topics, while users which co-occur the most are likely to share some interests and/or social relationship. Also, we assume that hashtags published by very similar users (with respect to shared interests) but which do not co-occur might still be related. The weights on *E*_*m*_ edges help to identify the more meaningful relations. Combining these relation types together also helps in strengthening a signal that might not emerge due to the restricted scope of the harvested network, relative to the entire Twitter network. The hashtag-user co-occurrence relation will be further exploited in step 13 for ranking purposes.

Based on the previous successful community detection for the network *G*_*C*_ and the explicit support for multiplex networks, Infomap [28] has been used in this step. Only meaningful communities with at least 30 nodes (an empirically determined threshold) and at least one hashtag (as we are interested in groups of related hashtags) are retained. Definition of each community topic takes place after the hashtag analysis in step 11, as some hashtags will be discarded at that stage as described below.

Hashtag analysis is performed in step 11, with the goal of filtering hashtags related to events and group those related to the same event. This step is performed one community at a time to prevent spurious correlations due to hashtag co-presence. The frequencies of hashtag usages among all the tweets in *UT*_*W*_ are re-sampled to the granularity of one day, then z-Score normalised. This provides zero-mean frequency distributions, from which negative-frequency values are set to zero to better consolidate the dataset.

**Fig. 4:**
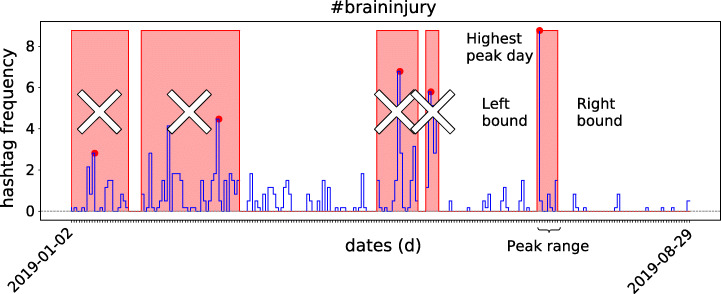
The “#braininjury” hashtag normalised usage frequency timeline sampled daily between the given dates. Each peak day (red dot) has a corresponding peak range (pink background). Only the highest peak and its range, are preserved, the rest is discarded

The goal of peak analysis is to determine whether it reflects a temporally isolated event in the time window. For each hashtag *h*, all the *peak*_*day* days which have a frequency peak value above the 90% percentile are identified^1^. This identifies left and right peak boundaries for *h*, denoted *lpb*(*h*) and *rpb*(*h*), respectively, as the largest contiguous interval of days in which the frequency is greater than 0 (with two days of tolerance). This interval defines the *peak rangepr*(*h*) = [*lpb*(*h*),*rpb*(*h*)], starting from *peak*_*day* itself. Each hashtag may have more than one associated peak. For each hashtag only the highest peaks is taken, if more than one have same height, then the peak with a larger interval is used. (hashtags with no peaks are discarded). An example is shown in Figure 4.

The highest degree hashtag, that is the hashtag with the highest number of edges connected to each *V*_*h*_ inside its community, is selected as the topic for the whole community.

Within each community, the Hashtag Temporal Correlation *HTC*(*h*_1_,*h*_2_) between pairs of hashtags *h*_1_,*h*_2_ within that community quantifies the amount of their temporal overlap, relative to their respective peak ranges *pr*(*h*_1_), *pr*(*h*_2_), and is defined as follows:

7 $$ {HTC}(h_{1}, h_{2}) = \frac{{overlap}({pr}(h_{1}), {pr}(h_{2}))}{{min}(count({pr}(h_{1})) , count({pr}(h_{2})))}.  $$

where *overlap*(*pr*(*h*_1_),*pr*(*h*_2_)) counts the number of overlapping days between the two peak ranges, and *count*(*pr*(*h*)) is the number of days within *h*’s peak range.

Recall from Section 3.1 that a context *C* = (*K*,[*t*_1_,*t*_2_],*s*) is defined as in (1) by a set *K* of hashtags, plus a time interval [*t*_1_,*t*_2_] (and possibly a geographical bounding box *s*). Here we use each group of two or more hashtags *h*_*i*_,*h*_*j*_ with *HTC*(*h*_*i*_,*h*_*j*_) > 0.5 to form the set *K* for a new *candidate context* *CC*.

Some resulting *K* may have a large number of hashtags, but this is undesirable since it brings noise and possible unwanted candidate context de-duplication.

For this reason, we have to decide what hashtag *h* ∈ *K* to keep for each candidate context, and we do so by ranking and keeping only the most important ones. First, we define

$$ w(u) = \frac{1}{R_{2}(u)}$$

to be the weight of *u*: in ranking the tags, the contribution of a user is inversely proportional to the user’s ranking (recall that the highest ranked hashtag has rank 1).

Now consider $C \in \mathcal {C}$ with tag set *K*. For each *h* ∈ *K*, let *U*(*h*) be the set of users who used *h* in *W*, and let *m*_*h*_ = |*U*(*h*)|.

For each candidate context $C \in \mathcal {C}$ we sort the hashtags *h* ∈ *K* with respect to $\text {h\_importance}(h) = {\sum }_{u \in U(h)} w(u)$ (descending). Only the top 5 hashtags are kept and the others are removed. The resulting candidate contexts are then merged together if they have the same *K*.

The time interval for the candidate context is set to include all peak ranges for those hashtags:

$$[t_{1}, t_{2}] = [\min \{{lpb}(h_{i})\}_{h_{i} \in K}, \max \{{rpb}(h_{i})\}_{h_{i} \in K}] $$

This procedure generates a set $\mathcal {C}$ of candidate contexts.

The last step 13 in the context discovery process involves ranking these context for relevance, as follows.

Recall that the tags used to define each $C \in \mathcal {C}$ are found in a set *UT*_*W*_ pertaining to window *W*. Consider a tag *h* and the set of users who used *h* within *W*. Those users will have been ranked in previous steps, as described in Section 3.2. We are going to use one of those rankings here, namely *R*_2_(*u*), to rank the tags found in *UT*_*W*_.

Using the number of users and their weights, we define multiple alternative ranking functions for *K*, and thus for *CC*, as follows.

8 $$ \textit{Ranking 1:} ~ {RC1}(K) = \frac{{\sum}_{h \in K} \frac{{\sum}_{u \in U(h)} w(u)}{m_{h}}}{|K|}  $$

This ranking is obtained by summing up all user weights for each tag in *K*, normalised by the number of users for that tag, and further normalised by the size of *K*.

9 $$ \textit{Ranking 2:} ~ {RC2}(K) = \frac{{\sum}_{h \in K} m_{h}} {|K|}  $$

Ranking 2 ignores the weights altogether, and only considers the total number of users that used each tag in *K*.

10 $$ \textit{Ranking 3:} ~ {RC3}(K) = \frac{{\sum}_{h \in K} \max_{u \in U(h)} w(u) } {|K|}  $$

Ranking 3 is similar to Ranking 1, but takes the sum of the largest weights for each tag.

11 $$ \textit{Ranking 4:} ~ {RC4}(K) = \frac{\max_{h \in K} \max_{u \in U(h)} w(u) } {|K|}  $$

Ranking 4 is similar to Ranking 3, but considers the overall max of the weights.

12 $$ \textit{Ranking 5:} ~ {RC5}(K) = \frac{{\sum}_{h \in K} m_{h} \max_{u \in U(h)} w(u) } {|K|}  $$

Finally, Ranking 5 multiplies the max weight for each tag by the number of users for that tag, and adds all the results.

The set $\mathcal {C}$ of all candidate contexts can be ranked according to any of these functions (and possibly more, user-defined, which can be easily added). Our system is only semi-automated, and these rankings are meant to provide support to expert users who will decide which of the contexts in $\mathcal {C}$ should be retained and be used in the next iteration of the entire pipeline.

## Evaluation

Greater sized contexts with more harvested tweets may increase the number of harvested users as well as better define them in terms of metrics. As an example, in [5], where the goal is to identify prominent information-sharing users during natural disasters, two datasets are used. The first one contains 152 402 tweets shared by 21 364, while the other consists of 44 330 tweets shared by 3 338 users. The first dataset is much greater than the second as it is used for training a supervised model, while the other is used as a validation set. In our approach, such big sized datasets are not required as our approach is based on the unsupervised method, thus not requiring a training phase. Also, the continuous harvesting nature of the framework, overcomes possible problems of poor user characterisations by repeatedly improving their definition with subsequent framework iterations.

Our evaluation aims at empirically measuring the relevance of the users and contexts discovered by the pipeline. Typical approaches for performing this type of evaluation rely on expert-generated ground truth. Such approaches, however, are vulnerable to the subjectivity of the experts, with the risk that the evaluation would be measuring the fit of the model to the specific experts’ own assessment of user instances’ relevance. In contrast, we follow an unsupervised approach with no *a priori* knowledge of user relevance. The goal of our study is to demonstrate the perceived value of our pipeline in creating a set of active database composed of online users that are ready to be mined, along with examples of candidate user ranking functions and of new contexts, discovered as described in the previous sections. In this approach, the value is evaluated based on human expertise, which comes into play in two specific phases. Firstly, to assess and validate the top-*k* user lists produced by these functions, and secondly, to assess which of the new contexts are *in-scope* and/or *in-focus*, as explained in detail below.

The pipeline is fully implemented in Python using Pandas and the NetworkX public libraries and is available on github^2^.

We evaluated the pipeline in action through two case studies, concerning (i) health awareness campaigns in the UK and (ii) events and initiatives around the COVID-19 crisis in Italy (spring 2020). We opted for topics that address health awareness, where activism of specialists and non-specialists can be frequently found [31]. For each case, the input to the framework is a list of manually selected on topic contexts, in order to discover top ranked activists and use them to discover new contexts, whose relevance is manually validated as explained earlier. Both case studies were performed on a single Azure node with standard commodity configuration. Note that we do not focus on system performance as all components operate in near-real time. One exception is Twitter content harvesting, which is limited by the Twitter API and requires approximately 2 hours per context.

The experiment design is the same for both case studies, and is therefore only presented in detail for the first one, here below.

### Case study 1: UK health campaigns

#### Contexts and networks

We have manually selected 25 contexts within the scope of health prevention campaigns in the UK, all occurring in 2018 and well-characterised using predefined hashtags. By campaigns, we address not only official ones (from governments) disseminated in the social networks, but also those ones started by citizens or other organisations in the context of social networks.

Due to limitations imposed by Twitter on the number of posts that can be retrieved within a time interval, only 200 tweets were retrieved from each context.

The framework, in terms of scalability, can handle sizeable network graphs derived from tweets harvesting as the involved metrics are of trivial complexity, and also distributed versions of Infomap algorithm exist [34]. The Twitter APIs, because of the severe usage quota limitations, may represent a problem in the case of greater implementations of the framework. This limit can be overcome by using multiple Twitter developer accounts as done in [5], where 5 hosts and 30 developers accounts where used. Table 1 lists the events along with key metrics for their corresponding user-user networks. To recall, *assortativity* measures how frequently nodes with a high degree are likely to connect with other nodes with a high degree (> 0) or with a low degree (< 0). Negative figures (mean: -0.22, std. dev.: 0.17) are in line with what is observed on the broader Twitter network [14]. The very small figures for density, defined as $\frac {\#edges }{{{\#nodes}} \cdot ({\#nodes} -1)}$ (mean: 0.004, std. dev.: 0.002), suggest very few connections exist amongst users within a context. This makes it difficult to detect meaningful communities, as described below, thus for some contexts the topological metrics are measured on the entire network as opposed to within each community. This view is also supported by the small average node degree (mean: 2.04, std. dev.: 0.46) and the ratio of strongly connected components to the number of nodes (mean: 0.98, std. dev. 0.02).

#### Community discovery

DEMON and Infomap produce significantly different communities in each network. DEMON identifies communities in only 48% of the networks, with an average of only 1.92 communities per network and a slightly negative (-0.28) average assortativity per community, in line with the average for their parent networks. Only the users who belong to one of those communities, about 6%, are added to the database. For the remaining 52% networks in which any community is detected, users’ in-degrees are calculated using the entire network, and all users are added to the database, for a total of 3 570 users being added to the database in our experiments using DEMON.

In contrast, Infomap provides meaningful communities for all networks. Those with less than three users are discarded, leaving 18.88 communities per network on average, with 8.5 users per community on average. When using Infomap, 3 567 users were added to the database (on average 253 users per network). The average assortativity across all communities is again slightly negative (-0.43). Table 2 compares the two approaches on the key metrics just discussed. On the basis of this comparison, we recommend using Infomap, which we have used for our evaluation.

**Table 2:** Comparing DEMON to Infomap for community detection

Metric	DEMON	Infomap
Fraction of networks with no communities	0.52	0.0
Number of communities per context (avg)	1.92	18.88
Fraction of network users added to the DB (avg)	0.06	0.59
Fraction of repeat users added to the DB across networks	0.28	0.37

#### Users discovery

**Fig. 5:**
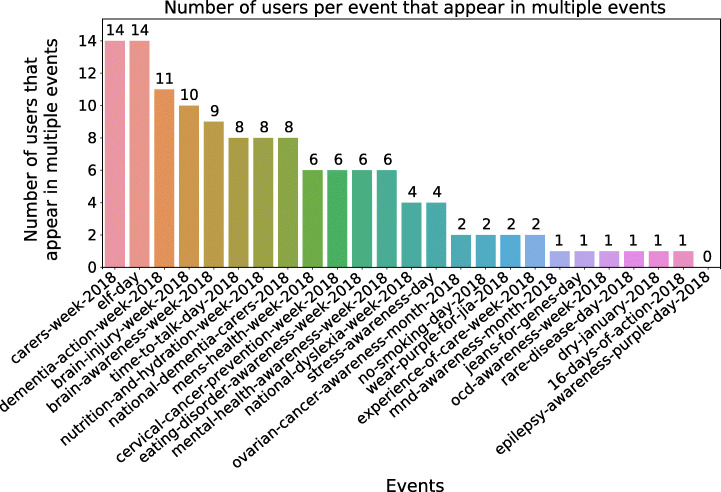
Number of repeat users for each context

Repeat users who appear in multiple contexts are particularly interesting as they provide a stronger signal of commitment to relevant contexts. Out of the total 3 567 users, 160 (4.5%) of them appear at least in two of the 25 contexts. After community detection, only 61 of these users are still seen as repeat users, while the remaining 99 are either removed altogether, or they only appear once. Of the 61, 57 appear twice, 2 appear three times, and 2 appear four times. Thus, only 1.6% of users appear more than once when communities with more than 3 users are considered, compared to the overall 4.5% of overall repeat users. Table 3 reports the top-10 repeated users along with their *Follower Rank*, and Figure 5 shows the number of repeat users per context. As the table is sorted first by number of the occurrences and then by *Follower Rank*, an indication of popularity, it is not surprising to find that top users include well-known names such as Mr. Hunt, who at the time of the events was Secretary of State for Health and Social Care in the UK, with *FR* = 1, and a number of associations and foundations active in the public healthcare space. More interesting are perhaps non-repeat users who emerge when ad-hoc ranking is applied to the database, as we illustrate next.

**Table 3:** Top-10 repeat users, amongst those who belong to a community

Username	Name	Follower rank	Participations
alzheimerssoc	Alzheimer’s Society	0.99	4
dementiauk	Dementia UK	0.98	4
mentalhealth	Mental Health Fdn	0.97	3
colesmillerllp	Coles Miller LLP	0.65	3
jeremy_hunt	Jeremy Hunt	1.0	2
nhsengland	NHS England	0.99	2
carersuk	Carers UK	0.95	2
rdash_nhs	RDaSH NHS FT	0.88	2
alzsocseengland	Alzheimer’s Society - South ...	0.64	2
mndassoc	MND Association	0.64	2

#### Users ranking

To demonstrate the potential value of the database, albeit on a small scale, we have tested three user ranking functions. As mentioned, this exercise aims to provide an objective grounding for engaging with experts on finding suitable operational definitions for specific user profiles. We consider good functions those that privilege individuals over organisations or businesses.

13 $$ \begin{array}{@{}rcl@{}} {Ranking 1:} ~ {R1}(u) & = \frac{1}{{\sum}_{u \in C} {IC}(u) + 1} \cdot {\sum}_{u \in C} {TF}(u) \end{array} $$

14 $$ \begin{array}{@{}rcl@{}} {Ranking 2:} ~ {R2}(u) & = \lvert {FR}(u) - 1 \rvert \cdot \left( {\sum}_{u \in C} {TA}(u) + {\sum}_{u \in C} {IC}(u)\right) \end{array} $$

15 $$ \begin{array}{@{}rcl@{}} {Ranking 3:} ~ {R3}(u) & = \lvert {FR}(u) - 1 \rvert \cdot \left( {\sum}_{u \in C} {TA}(u) + \frac{1}{{\sum}_{u \in C} {IC}(u) + 1}\right) \end{array} $$

All the three functions (13), (14) and (15) consider every community *C* where the given user *u* have appeared (*u* ∈ *C*) across all the contexts. Function (13) is designed to promote users who are at the “fringe” of their community, while giving credit to generic on-topic activities during the contexts. To achieve this, *Topical FocusTF* is used as a positive contribution, while a large in-degree *IC* reduces the score. In contrast, function (14) penalises user popularity, i.e., by using the complement of *Follower Rank* *FR*, while rewarding prominence inside communities (in-degree *IC*) and information spreading by also considering shared links (*Topical AttachmentTA*). Function (15) combines ideas from both (14) and (13).

**Fig. 6:**
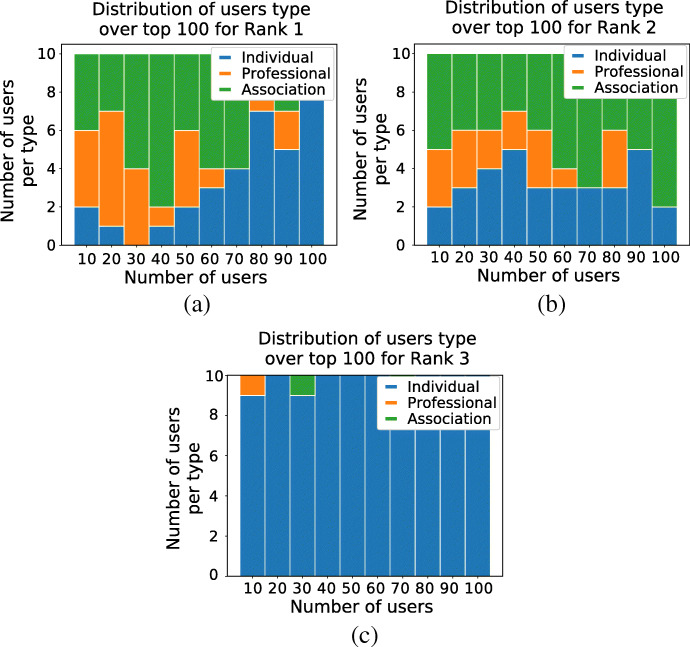
Distribution of user types for top-100 users and for each ranking function

The top-10 users for each ranking are reported in Table 4. To appreciate the effects of these functions, we have manually labelled the top-100 user profiles for each of the rankings, using a broad type classification as *individuals* as opposed to *institutional players* (associations, public bodies), or *professionals*. The fractions of on-topic users are 86%, 83%, and 38% for (13), (14), and (15) respectively. Importantly, (15) identifies more individuals than institutions and professionals (96%) than (14) and (13), both at 33%p. Also, repeat users are given a higher score in both rankings. Users with *FR*(*u*) = 0 and ${min\_max(\lvert Tweets (u)\rvert ) < 0.005}$ are considered not active and have been assigned lowest score. Figure 6 shows the distribution of user types within the top-100 users for each of the three rankings, broken down into 10 users bins. We can see that individuals dominate in (15), and are fewer but emerge earlier in the ranks when (14) is used.

**Table 4:** Top-10 ranked users for ranking functions (13) and (14) and (15), with indication of whether the user is on-topic/off-topic and individual vs association/professional

	Ranking 1			Ranking 2			Ranking 3		
#	User	On-topic	Individual	User	On-topic	Individual	User	On-topic	Individual
1	homesnutrition	X		johnneustadt	X		johnneustadt	X	
2	ficajones	X	X	jo_millar27	X	X	solutions777	X	X
3	helenvweaver	X	X	hatchbrenner			kingste29344921	X	X
4	spriggsnutri	X		nchawkes	X	X	daisylu1964		X
5	critcarelthtr	X		moz0373runner	X	X	zakariamarsli	X	X
6	danielleroisin_	X	X	aimsonhealth	X	X	meowaaaaaa		X
7	mynameisandyj	X	X	wordsharkv5		X	vecta67		X
8	fionaliu92	X	X	fullcircle_play	X		cosfordfamily1	X	X
9	ldpartnership	X		qsprivatehealth	X		hayleycorriganx		X
10	milaestevam1		X	socialissp			jhbrasfie		X

Such categories are useful to evaluate the ranking functions

#### Context harvesting

We now evaluate the strategy presented in Section 5.3 for discovering new contexts, which implements the second phase of the pipeline (refer to Figure 1). Although this phase “closes the loop” to enable a new iteration of users discovery, here we only demonstrate one instance of the complete loop, by showing how new contexts can be found from the set of users we discovered in the previous part of the evaluation.

We looked for new contexts within an 8-months window (2019-01-01 and 2019-08-29) after the initial user discovery. This interval follows and does not overlap with the time interval where our initial 25 contexts were seeded (Section 6), and which ranged from 2018-01-01 (“Dry january”, Table 1) to 2018-12-12 (“Elf day”, Table 1).

For the purpose of this experiment, we selected the top 1 000 out of a total of 3 567 ranked users (Section 6.1.3), and we harvested up to 3 200 of their tweets from their timelines (due to Twitter limitation API) in the aforementioned time interval.

**Fig. 7:**
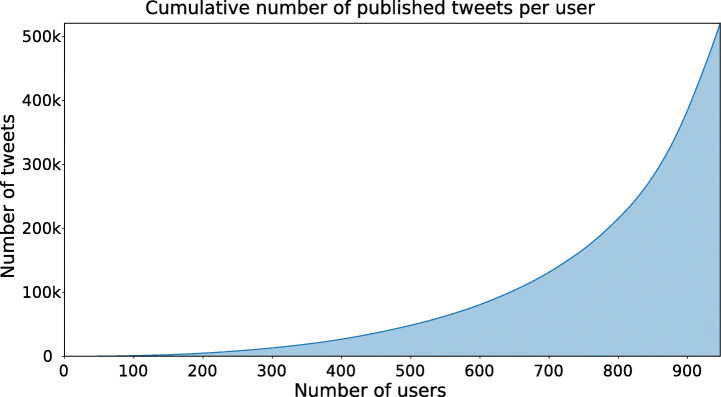
Cumulative sum of tweet count by the top ranked users from 2019-01-01 to 2019-08-29

This resulted in a total of 520 861 tweets (Figure 7), considering that 59 users had become inactive. The user posting behaviour is characterised by an a high variance in the tweet post count, ranging from a minimum of 1 to a maximum of 3 200 tweets, hitting the Twitter API limit.

##### Multiplex network generation

A multiplex network as described in Section 5.3 was generated using all users, either author or mentioned, and all hashtags from each harvested tweet. The network consists of 52 601 nodes and 211 235 edges. Of these, main Hashtag-Hashtag network has 51 676 nodes and 104 595 edges (98.24% of the nodes and the 49.52% of the edges), while the much smaller User-User network has 925 nodes and 2 161 edges. The two networks are connected by 104 479 edges, or 49,52% of the total.

**Fig. 8:**
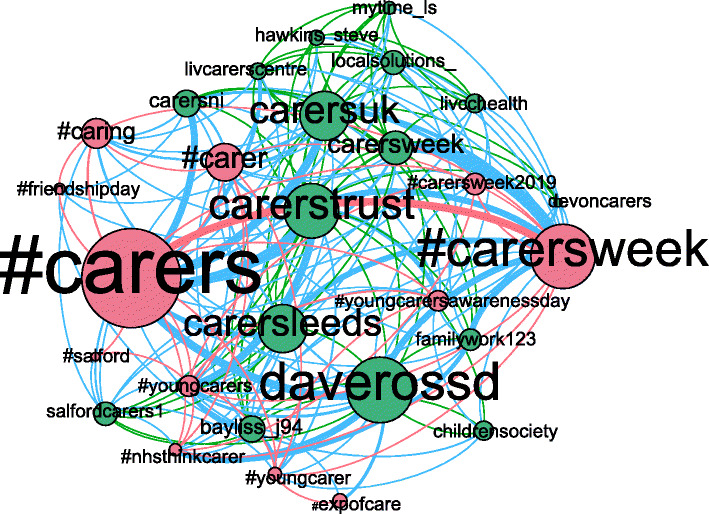
Example of the network of a community found after applying Infomap, from the UK healthcare case. A community in our process represents a topic, which contains some candidate contexts. Nodes, represented by circles, are proportional in size to the degree. It can be Twitter users (green) or hashtags (red). Edges, represented by lines, are proportional in size to the weight, and can either be user-user (green), hashtag-hashtag (red) or user-hashtag (blue) relations

In the next phase, the Infomap algorithm detected 367 communities of nodes and hashtags. This process groups nodes, either hashtags or users, which are related to the same topic, and also reduces the network size by only keeping communities with at least 30 nodes and at least an hashtag node (an example of such discovered communities is shown in Figure 8).

These communities exhibit low assortativity (mean: -0.59, std. dev.: 0.28) and high density (mean: 0.64, std. dev.: 0.39), meaning that the networks are characterised by highly connected nodes and central node hubs. The majority of the communities have a small number of nodes (mean: 4.11, std. dev.: 4.82) and edges (mean: 9.55 std. dev.: 27.47), while fewer (71 communities) have more nodes than the average.

After pruning the communities with fewer than 30 nodes, we are left with a smaller overall network of 1 903 nodes and 39 597 edges, a reduction in size of 96.38% and 81.25% of nodes and edges, respectively. At this point in the process, the User-User network now has 417 nodes and 1 184 edges, and the Hashtag-Hashtag network has 1 486 nodes and 17 057 edges (78.09% and 43.08% of the nodes and edges, respectively). The two networks are still connected by the majority of the edges (21 356, or 53.93% of the total).

##### Peak analysis

Peak analysis consists of two steps. In the first step just described, we determine whether a tag belongs to an isolated event, or if it is a common topic not related to any particular event, by looking at the temporal distribution of every hashtag. The second step focuses on the relationship between any two hashtags, by observing their temporal correlation (7), to determine when two or more hashtags are related to the same event. Note that there is a possibility of finding spurious temporal correlation between two hashtags, i.e., two hashtags which have the highest peak at the same moment, but are related to completely different topics. This, however, is mitigated by the community detection process, which groups users and hashtags with respect to a common topic, indeed this step is performed only between hashtags belonging to the same community.

Peak analysis is important once it helps to understand whether a hashtag belongs to an event or not and whether it correlates with any other hashtag. It is performed for each hashtag within each community. For instance, Figure 4 presents the results of our analysis, composed of 2 385 peaks with 301 communities (out of 367) containing at least one peak.

The average number of hashtag usages (z-score normalised) for a single peak is 2.04 (std. dev. 4.96), with the average peak duration of 3.13 days (std. dev. 6.07). In both cases, a high variance characterises these quantities. A total of 1 184 hashtags have at least one peak, whereas the peak distribution is uneven (mean: 2.01, std. dev.: 2.10), having a maximum of 23 peaks per hashtag. The peaks distribution among communities is also uneven, with an average of 6.42 peaks with a standard deviation of 9.15.

As it turns out, the majority of the hashtags is unrelated to all others (mean of related hashtags = 1.16, std. dev. 0.61). Fewer hashtags will have a relationship with others, however. To illustrate, Table 5 presents the longest groups of hashtags found in this step).

**Table 5:** Top-10 of the bigger groups of correlated hashtags coupled with the related topic

#	Topic	Correlated hashtags
1	#uk	#borisjohnson, #eds, #maimes, #me, #pacetrial, #pwme
2	#internationalnursesday	#ind2019, #internationalnursesday, #nursesday, #nursesday2019, #teamwhh
3	#uk	#eds, #maimes, #me, #pacetrial, #pwme
4	#neuroscience	#fens, #frm2019, #training
5	#dementia	#daw2019, #dementiaactionweek, #dementiaawarenessweek
6	#carers	#carersweek, #carersweek2019, #caring
7	#braininjury	#abiweek, #fatigue, #hatsforheadway
8	#genderequality	#metoo, #thepowerof, #wd2019
9	#mentalhealthawarenessweek	#mentalhealthawarenessweek, #mhaw19
10	#epilepsy	#purpleday, #seizures

The correlated hashtags are the core of the new context candidates. We can appreciate how close is the topic chosen to the associated correlated hashtag list, and how most of the hashtags in the same group relate to the same context

##### Context ranking and labelling.

**Fig. 9:**
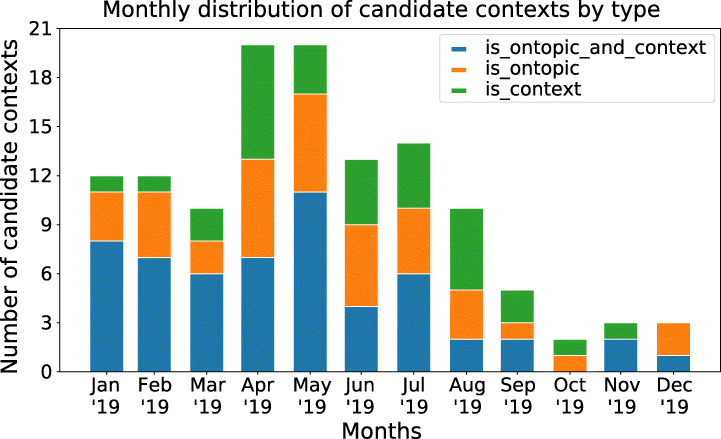
Monthly distribution of significant candidate contexts by type. Note that the last 4 months contain less candidate contexts, this is due to hitting the 3200 most recent tweets limit from the user timeline Twitter API

**Fig. 10:**
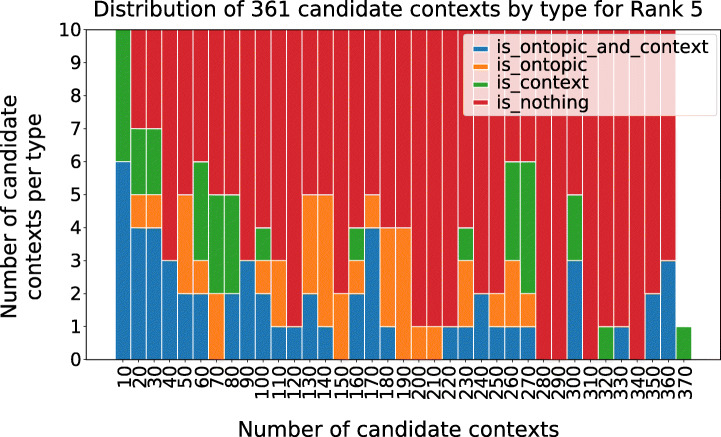
Distribution of the candidate context by type sorted by Rank 5 12. Note how on-topic candidate contexts, which are actual contexts, are ranked higher (blue). Of those, 87 are actual contexts, 93 are in topic and, out of those, 56 are both (“is_ontopic_and_context”)

Each of the hashtags groups forms a candidate context. As anticipated, the final selection of new contexts from this candidate sets is left to experts, and involves labelling each candidate as either on or off-topic, and on-context vs off-context (visually temporally distributed in Figure 9). To support this final phase and help the experts focus on a few contexts, a ranking is applied which favours candidate contexts which are likely to be both on-topic with UK awareness campaigns (Section 6.1.1) and that respond to our operational definition of contexts (1). Out of the five ranking functions introduced in Section 5.3, here we demonstrate Ranking 5, (Eq. 12), which shows experimentally a prominence of good candidate contexts towards the top (Figure 10). These final top context are shown in Table 6.

**Table 6:** Top-10 ranked candidate contexts for ranking function 5 (12), with indication of whether the candidate context is on-topic/off-topic and context vs non-context

	Candidate Context Ranking 5					
#	Topic	Hashtags list	Start date	End date	On-context	On-topic
1	#valentinesday	#valentinesday	02-04	02-18	X	
2	#easter	#easter	04-01	04-24	X	
3	#internationalwomensday	#balanceforbetter, #internationalwomensday, #iwd19, #iwd2019	03-02	03-13	X	X
4	#mentalhealthawarenessweek	#bebodykind, #mentalhealthawarenessweek	05-06	05-21	X	X
5	#worldbookday	#worldbookday	03-05	03-08	X	
6	#cancer	#worldcancerday	01-30	02-07	X	X
7	#leeds	#bluemonday	01-21	01-21	X	X
8	#mothersday	#fathersday	06-10	06-18	X	
9	#mentalhealth	#timetotalk, #timetotalkday	01-30	02-08	X	X
10	#nhs	#nottinghamshire, #volunteers, #volunteersweek, #volunteersweek2019	05-28	06-12	X	X

Such categories are useful to evaluate the candidate context ranking functions

### Case study 2: Italian COVID-19 related initiatives

The experiments for this second case study follow closely the structure just outlined in the previous section. For this case, we have manually selected 24 contexts within the scope of COVID-19 awareness campaigns in Italy, all occurring at the end of February and March 2020. Key metrics for the user-user networks are presented in Table 7. Like in our previous case, low density (mean: 0.035, std. dev.: 0.1), compounded by the small average node degree (mean: 2.32, std. dev.: 0.70) and the ratio of strongly connected components to the number of nodes (mean: 0.96, std. dev. 0.01), suggest very few connections amongst users within a context.

**Table 7:** List of contexts used in the experiments along with network metrics (Note: “Ero in corsia” assortativity computation results in a division by 0, as the only 2 present nodes have the same degree (they are connected to each other)

Context name	Period (2020)	Nodes	Edges	Density	Avg degree	Assortativity
Musei chiusi musei aperti	02-25 / 03-31	192	341	0.009	3.6	− 0.1
Non farti influenzare	02-27 / 03-31	24	41	0.074	3.4	0.9
Confcommercio ce	02-27 / 03-31	80	132	0.021	3.3	− 0.3
Italia non si ferma	02-28 / 03-31	177	169	0.005	1.9	− 0.4
Regioni non si fermano	02-28 / 03-08	199	163	0.004	1.6	− 0.2
Solidarieta digitale	02-28 / 03-31	209	285	0.007	2.7	− 0.4
Cultura non si ferma	03-01 / 03-31	180	195	0.006	2.2	− 0.2
Musica non si ferma	03-01 / 03-31	161	180	0.007	2.2	− 0.4
Io resto a casa	03-07 / 03-31	294	205	0.002	1.4	− 0.1
Allenati a casa	03-08 / 03-31	105	105	0.01	2.0	− 0.4
Suono da casa	03-08 / 03-31	159	148	0.006	1.9	− 0.3
Campagna non si ferma	03-09 / 03-31	85	114	0.016	2.7	− 0.2
Spesa a domicilio	03-10 / 03-31	77	100	0.017	2.6	0.1
Leggo da casa	03-10 / 03-31	217	220	0.005	2.0	− 0.2
Ripartiamo insieme	03-10 / 03-31	98	135	0.014	2.8	0.2
Avis campaign	03-11 / 03-31	174	176	0.006	2.0	− 0.5
Voi restate a casa	03-12 / 03-31	208	210	0.005	2.0	− 0.5
Insieme per il paese	03-13 / 03-31	100	195	0.02	3.9	− 0.5
Dl curaitalia	03-16 / 03-31	217	199	0.004	1.8	− 0.4
Csv italia	03-17 / 03-31	23	21	0.041	1.8	− 0.6
La cultura in casa	03-18 / 03-31	149	184	0.008	2.5	− 0.2
Poesie in quarantena	03-19 / 03-31	105	105	0.01	2.0	− 0.7
Aiutiamo gli eroi	03-26 / 03-31	29	35	0.043	2.4	− 0.7
Ero in corsia	03-27 / 03-31	2	1	0.5	1.0	NaN

We have used Infomap for community discovery, which produced 10.74 communities per network and 12.36 users per community on average, resulting in 2 387 users being added to the database (on average 136 users per network).

We found nearly twice as many repeat users (9.09%) as in the previous case, of which 133 are left following community detection, that is 5.57% of users appear more than once when communities with more than 3 users are considered. Of these, 116 appear twice, 12 appear three times, and 3 appear four times, 1 appears five times, and 1 appears six times.

**Fig. 11:**
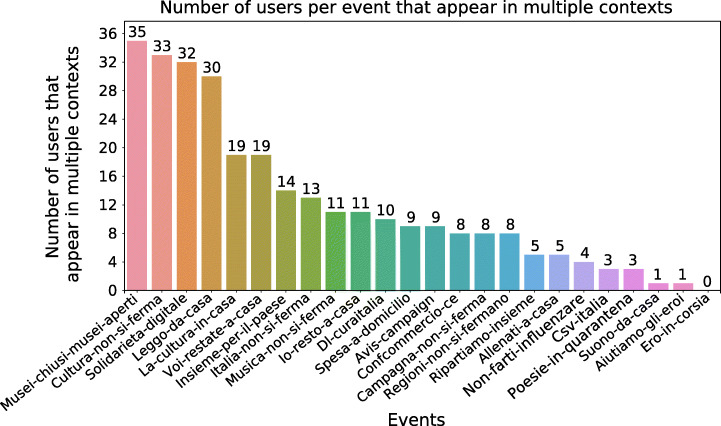
Number of repeat users for each context

The top-10 repeat users along with their *Follower Rank* are shown in Table 8, and the numbers of repeat users per context are in Figure 11. In this study, we found notable personalities amongst the top users, such as Giuseppe Conte, the Prime Minister of Italy at the time of the events, with *FR* = 1. Among the top users, we also found accounts of several institutional and cultural associations.

**Table 8:** Top-10 repeat users, amongst those who belong to a community

Username	Name	Follower rank	Participations
youtube	YouTube at	1.0	6
marino29b	marino29b	0.66	5
repubblica	Repubblica	1.0	4
comunemi	Comune di Milano	1.0	4
_mibact	MiBACT	0.97	4
mise_gov	MISE	1.0	3
giuseppeconteit	Giuseppe Conte	1.0	3
sanremoanchenoi	Sanremo anche noi	0.98	3
artdielle	Arturo D.L.	0.83	3
casalettori	Casa Lettori	0.69	3
adelestancati	adele stancati	0.64	3

**Fig. 12:**
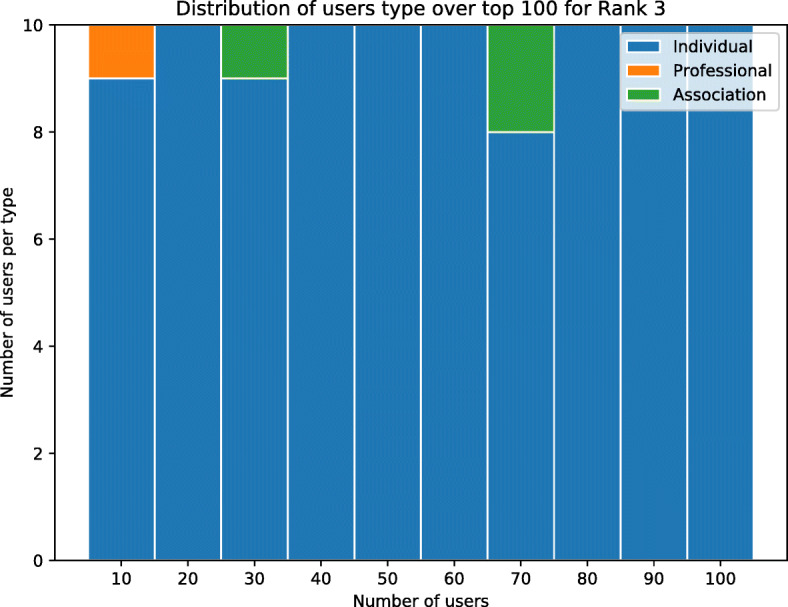
Distribution of user types for top-100 users and for each ranking function. Rank 3: 97 are individuals, 5 are professionals, 1 are associations

Using the same ranking functions as in the first case study, we found the top-10 users for each ranking as listed in Table 9. Following manual labelling of the top-100 profiles, 48% of users are on-topic. Figure 12 shows the full breakdown.

**Table 9:** Top-10 ranked users for ranking function (15), with indication of whether the user is on-topic/off-topic and individual vs association/professional

	Ranking 3		
#	User	On-topic	Individual
1	ileniacostanza2		X
2	gabbianorp		X
3	mattecurvasudm1		X
4	laura40805025	X	X
5	aleseminati	X	X
6	lapazzariel	X	X
7	robbi00698088		X
8	losateresa	X	X
9	andreal79733639	X	X
10	elisanucera		X

#### Users ranking

Finally, we tested the context harvesting capability of the pipeline, within a one-month window (2020-04-01 and 2020-04-30, as the crisis is still ongoing at the time of writing) after the initial user discovery. Again, this interval follows and does not overlap with the time interval where our initial 24 contexts were seeded (Section 6), and which ranged from 2020-02-25 (“Musei chiusi musei aperti”, Table 7) to 2020-03-31 (“Ero in corsia”, Table 7).

**Fig. 13:**
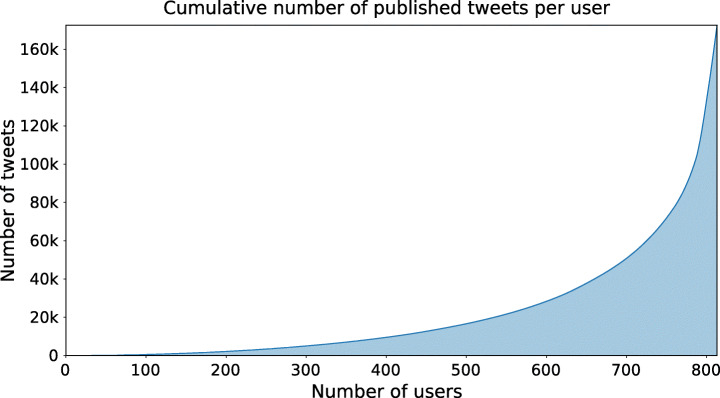
Cumulative sum of tweet count by the top ranked users from 2020-04-01 to 2020-04-30

We harvested 3 200 tweets for each of 2 387 users, resulting in a total of 209 888 tweets (Figure 13) with high variance, ranging between 1 to 3 190 tweets.

##### Multiplex network generation.

**Fig. 14:**
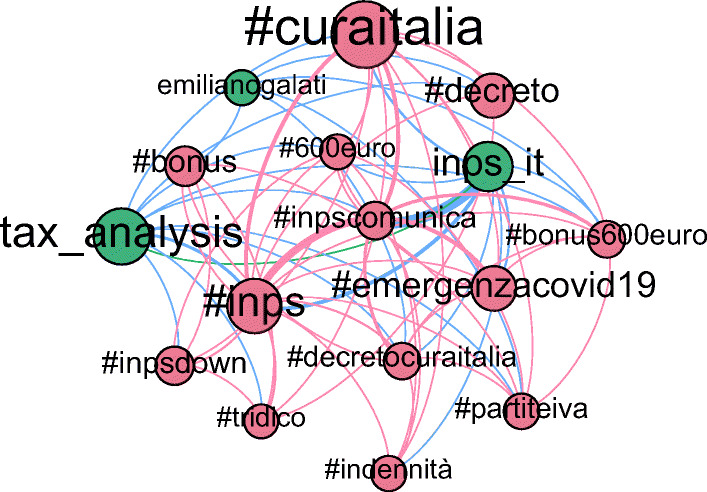
Example of the network of a community found after applying Infomap, from the COVID-19 Italy case

In this case, the multiplex network consists of 23 281 nodes and 125 326 edges, of which 22 366 nodes and 59 629 edges (96.07% of the nodes and the 47.58% of the edges) are part of the Hashtag-Hashtag network, and the rest (915 nodes and 4 166 edges) is the User-User network. The two networks are connected by 61 531 edges, or 49,09% of the total, which incidentally is almost exactly identical to the first case. 223 communities of nodes and hashtags are generated by the Infomap algorithm, resulting in a reduced network of 1 241 nodes and 31 629 edges, a reduction in size of 94.67% and 74.75% of nodes and edges, respectively (an example of such discovered communities is shown in Figure 14).

##### Peak analysis.

**Fig. 15:**
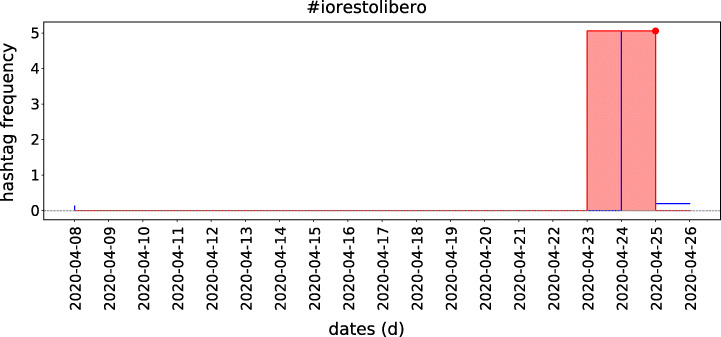
The “#iorestolibero” hashtag normalised usage frequency timeline sampled daily between the given dates. Each peak day (red dot) has a corresponding peak range (pink background)

For this case, our algorithm detected 767 peaks, with 135 communities (out of 223) containing at least one peak (Figure 15).

Compared with the 2.62 hashtags / peak (duration 3.13 days) for the first case study, here we have 1.62 hashtags / peak with average peak duration of 6.77 days. A total of 634 hashtags have at least one peak, with a maximum of 4 peaks per hashtag. There are also more related tags (2.45 on average) than in the first case, with the longest groups of hashtags presented in Table 10.

**Table 10:** Top-10 of the bigger groups of correlated hashtags coupled with the related topic

#	Topic	Correlated hashtags
1	#coronavirus	#coronavirus, #covid19, #covid_19, #covid-19, #fase2
2	#coronavirus	#coronavirus, #covid19, #covid_19, #covid-19, #pasqua
3	#coronavirus	#coronavirus, #covid19, #covid_19, #fase2, #italia
4	#25aprile	#25aprile, #liberazione, #25aprile2020, #festadellaliberazione, #bellaciao
5	#25aprile	#25aprile, #liberazione, #25aprile2020, #bellaciao, #iorestolibero
6	#25aprile	#25aprile, #25aprile2020, #festadellaliberazione, #bellaciao, #resistenza
7	#conte	#conte, #mes, #salvini, #governo, #lega
8	#iorestoacasa	#iorestoacasa, #artyouready, #andràtuttobene, #granvirtualtour, #viaggioinitalia
9	#iorestoacasa	#iorestoacasa, #andràtuttobene, #granvirtualtour, #iorestocasa, #museichiusimuseiaperti
10	#iorestoacasa	#iorestoacasa, #granvirtualtour, #iorestocasa, #museichiusimuseiaperti, #lartetisomiglia

##### Context ranking and labelling.

**Fig. 16:**
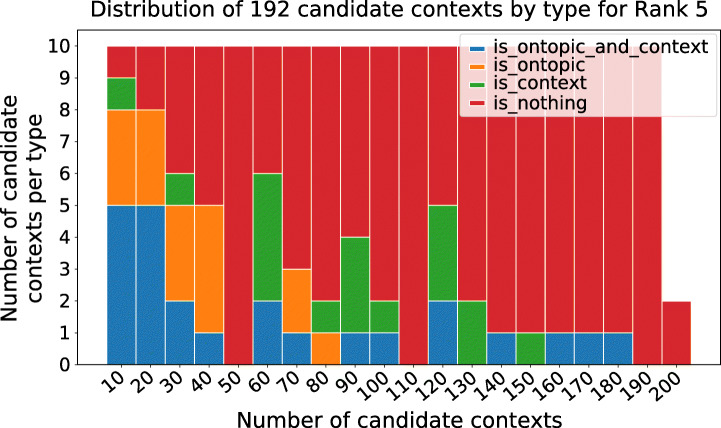
Distribution of the candidate context by type sorted by Rank 5 12: 41 are actual contexts, 40 are in topic and, out of those, 24 are both (“is_ontopic_and_context”)

**Fig. 17:**
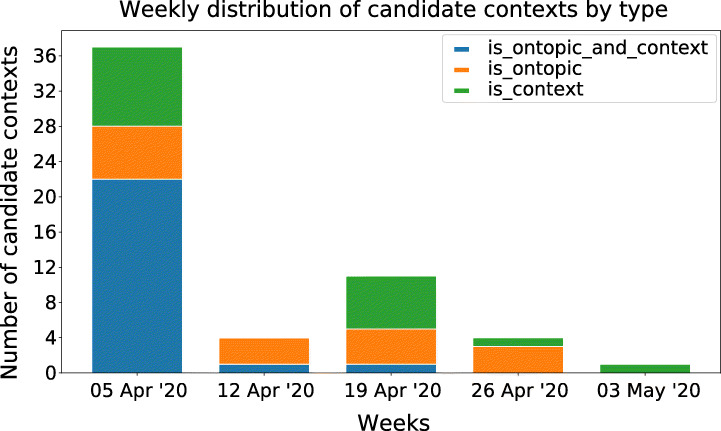
Monthly distribution of significant candidate contexts by type. Note that the last weeks contain fewer candidate contexts. It happens due to the fact that in such a short temporal horizon most contexts started at the beginning of the month or were already happening before

Finally, Figure 16 and Table 11 show the top candidate contexts likely to be both on-topic with COVID-19 campaigns in Italy, and the final top contexts after manual labelling, respectively. Cadidate contexts are visually temporally distributed in Figure 17).

**Table 11:** Top-10 ranked candidate contexts for ranking function 5 (12), with indication of whether the candidate context is on-topic/off-topic and context vs non-context

	Candidate Context Ranking 5					
#	Topic	Hashtags list	Start date	End date	On context	On topic
1	#coronavirus	#coronavirus, #covid19, #covid_19, #covid-19, #fase2	04-01	04-29	X	X
2	#coronavirus	#coronavirus, #covid19, #covid_19, #covid-19, #pasqua	04-01	04-29	X	X
3	#coronavirus	#coronavirus, #covid19, #covid_19, #fase2, #italia	04-01	04-29	X	X
4	#25aprile	#25aprile, #liberazione, #25aprile2020, #festadellaliberazione, #bellaciao	04-18	04-28	X	
5	#25aprile	#25aprile, #liberazione, #25aprile2020, #bellaciao, #iorestolibero	04-18	04-28	X	
6	#iorestoacasa	#iorestoacasa, #viaggioinitalia, #museichiusimuseiaperti	04-01	04-29	X	X
7	#25aprile	#25aprile, #25aprile2020, #festadellaliberazione, #bellaciao, #resistenza	04-18	04-28	X	
8	#milano	#milano	04-01	04-29		
9	#conte	#conte, #mes, #salvini, #governo, #lega	04-01	04-29		X
10	#iorestoacasa	#iorestoacasa, #andràtuttobene, #museichiusimuseiaperti, #lartetisomiglia	04-01	04-29	X	X

## Conclusions and lessons learnt

Motivated by the need to find an operational definition of “online activists” grounded in well-established network and user-activity metrics, we have designed a Twitter content processing pipeline for progressively harvesting Twitter users based on their engagement with online socially-minded events, or campaigns, which we have called *contexts*.

The pipeline yields a growing database of user profiles along with their associated metrics, which can then be analysed to experiment with user-defined user ranking criteria. The pipeline is designed to select promising candidate profiles, but the approach is unsupervised, i.e., no manual classification of example users is provided. We have validated the pipeline on two real and recent life case studies, UK Health campaigns and social campaigns associated with the fight against COVID-19 in Italy.

Although both topics address health prevention campaigns in particular countries, it is important to note that the first topic is more generic, covering any campaign for health prevention. On the other hand, the second topic is more specific, addressing the prevention of a specific disease (COVID-19) in the context of an ongoing pandemics. We found similar behaviours among the activists of both topics. These findings strengthen our perception that online activism is a continuous and recurrent practice in social networks, despite the urgency and complexity of the topic. This suggests that, using our approach, we may be able to harvest on-line activists in the context of other topics addressing public interest.

The design of the pipeline shows that useful harvesting of interesting users can be accomplished within the limitations imposed by Twitter on its APIs. The next challenge is to completely automate the discovery of new contexts so that the pipeline may continuously add new and update users in the database.

## Footnotes

1 This is done using: https://docs.scipy.org/doc/scipy/reference/generated/scipy.signal.find_peaks.html

2 https://github.com/flaprimo/twitter-network-analysis
